# ErbB2 (HER2)-CAR-NK-92 cells for enhanced immunotherapy of metastatic fusion-driven alveolar rhabdomyosarcoma

**DOI:** 10.3389/fimmu.2023.1228894

**Published:** 2023-08-18

**Authors:** Catrin Heim, Laura M. Moser, Herman Kreyenberg, Halvard B. Bonig, Torsten Tonn, Winfried S. Wels, Elise Gradhand, Evelyn Ullrich, Michael T. Meister, Marian Groot Koerkamp, Frank C. P. Holstege, Jarno Drost, Jan-Henning Klusmann, Peter Bader, Michael Merker, Eva Rettinger

**Affiliations:** ^1^ Goethe University Frankfurt, Department of Pediatrics, Division for Stem Cell Transplantation, Immunology and Intensive Care Medicine, Frankfurt am Main, Germany; ^2^ German Cancer Consortium (DKTK), Partner Site Frankfurt am Main, a Partnership Between DKFZ, University Hospital and Georg-Speyer-Haus, Frankfurt am Main, Germany; ^3^ Frankfurt Cancer Institute (FCI), Goethe University, Frankfurt am Main, Germany; ^4^ Universitäres Centrum für Tumorerkrankungen (UCT), Frankfurt am Main, Germany; ^5^ Department of Cellular Therapeutics/Cell Processing, Institute for Transfusion Medicine and Immunotherapy, Goethe University, Frankfurt am Main, Germany; ^6^ Division of Hematology, Department of Medicine, University of Washington, Seattle, WA, United States; ^7^ Experimental Transfusion Medicine, Faculty of Medicine Carl Gustav Carus, TU Dresden, Dresden, Germany; ^8^ German Cancer Consortium (DKTK), Partner Site Dresden, Dresden, Germany; ^9^ Georg-Speyer-Haus, Institute for Tumor Biology and Experimental Therapy, Frankfurt am Main, Germany; ^10^ Dr. Senckenberg Institute of Pathology, University Hospital Frankfurt, Frankfurt, Germany; ^11^ Experimental Immunology, Department for Children and Adolescents, University Hospital Frankfurt, Goethe University, Frankfurt, Germany; ^12^ Princess Máxima Center for Pediatric Oncology, Utrecht, Netherlands; ^13^ Oncode Institute, Utrecht, Netherlands; ^14^ Center for Molecular Medicine, UMC Utrecht and Utrecht University, Utrecht, Netherlands; ^15^ Goethe University Frankfurt, Department of Pediatrics, Frankfurt am Main, Germany

**Keywords:** ERBB2 (HER2/neu), rhabdomyosarcoma, chimeric antigen receptor, xenograft, cancer immunotherapy

## Abstract

**Introduction:**

Metastatic rhabdomyosarcoma (RMS) is a challenging tumor entity that evades conventional treatments and endogenous antitumor immune responses, highlighting the need for novel therapeutic strategies. Applying chimeric antigen receptor (CAR) technology to natural killer (NK) cells may offer safe, effective, and affordable therapies that enhance cancer immune surveillance.

**Methods:**

Here, we assess the efficacy of clinically usable CAR-engineered NK cell line NK-92/5.28.z against ErbB2-positive RMS *in vitro* and in a metastatic xenograft mouse model.

**Results:**

Our results show that NK-92/5.28.z cells effectively kill RMS cells *in vitro* and significantly prolong survival and inhibit tumor progression in mice. The persistence of NK-92/5.28.z cells at tumor sites demonstrates efficient antitumor response, which could help overcome current obstacles in the treatment of solid tumors.

**Discussion:**

These findings encourage further development of NK-92/5.28.z cells as off-the-shelf immunotherapy for the treatment of metastatic RMS.

## 1 Introduction

Rhabdomyosarcoma (RMS) is the most prevalent type of soft tissue sarcoma in children (1–3). Adolescents and young adults over the age of 9 with advanced metastatic, primary refractory or relapsed (r/r), alveolar subtype RMS involving the bone or bone marrow (BM) and/or exhibiting fusion-positive (PAX3/7-FOXO1) characteristics have a particularly poor prognosis. These patients are likely to succumb to their disease within an average of two years following diagnosis (2, 4–6). Even for those who survive, long-term debilitating effects can result from substantial treatment-related toxicity, such as cardiotoxicity and nephrotoxicity.

Developing new therapeutic strategies to overcome tumor resistance remains a critical unmet need for these heavily pretreated patients. Chimeric antigen receptor (CAR)-engineered cells, which target and inhibit the proliferation and spread of tumor cells, are revolutionizing the treatment of r/r malignancies. However, this therapy can also lead to severe, albeit treatable, side effects, such as cytokine release syndrome (CRS), immune effector cell-associated neurotoxicity syndrome (ICANS), and secondary hemophagocytic lymphohistiocytosis/macrophage activation syndrome (7).

The lack of suitable CAR target antigens, as well as the immunosuppressive nature of the tumor microenvironment (TME), and advanced-stage disease, renders CAR-T cells nonresponsive or exhausted against many solid tumors (8, 9). However, targeting ErbB2 (HER2) in metastatic alveolar RMS (aRMS) (10–12) by the patient’s autologous CAR-T cells appeared to be safe and feasible in a phase I/II clinical trial (13), with a complete response observed in a case report with one child (13, 14).

Here, we employed a molecularly and functionally well-defined clonal derivative of the natural killer (NK) cell line NK-92 (NK-92/5.28.z) as effector cells (15–17), carrying an ErbB2-specific second-generation CAR with a composite CD28-CD3ζ signaling domain. Compared to other CAR-NK or CAR-T-cell products, good manufacturing practice (GMP) production of the off-the-shelf NK-92/5.28.z cell product in clinically relevant doses is affordable and less complex or labor-intensive (17). Intralesional therapy with third party, off-the-shelf NK-92/5.28.z cells is currently being explored in a phase I clinical trial (NCT03383978) in patients with recurrent ErbB2-positive glioblastoma (16, 18).

In a proof-of-concept analysis, we previously demonstrated specific ErbB2-CAR-mediated recognition and killing of ErbB2-expressing RMS cell lines by NK-92/5.28.z cells *in vitro* (19). Here, recognition of target cells triggered surface expression of CD107a and degranulation of NK-92/5.28.z cells, but not of parental NK-92 cells. The heterogeneous expression of tumor antigens, hypoxia, and the immunosuppressive TME of solid tumors adversely impact the accessibility, infiltration, stimulation, activation, and persistence of conventional CAR-engineered T cells at the tumor site (20). The cancer immune surveillance of NK-92/5.28.z cells is triggered by the anti-ErbB2-targeted CAR and germline-encoded cell surface receptors, as well as by supporting adoptive antitumor immune responses of T cells in the TME (21), suggesting homing to, infiltration into, and persistence of NK-92/5.28.z cells at RMS tumor sites.

To further develop the NK-92/5.28.z cell therapy towards clinical application as a consolidation treatment for children and young adults with r/r, metastatic, ErbB2-positive RMS tumors after re-induction radiotherapy/chemotherapy, we performed detailed preclinical *in vitro* and *in vivo* safety and efficacy analyses of NK-92/5.28.z cells in clinically relevant, experimental metastasis models, including a human (*PAX3-FOXO1*) aRMS xenograft model.

## 2 Materials and methods

### 2.1 Cell lines and cell culture

The NK-92 cell line was derived from a 50-year-old patient with non-Hodgkin´s lymphoma (22). NK-92/5.28.z cells were generated as a single-cell clone by lentiviral transduction of NK-92 cells using the pS-5.28.z-W CAR vector as reported previously (15). This vector encodes a codon-optimized second-generation CAR harboring the ErbB2-specific scFv (FRP5) antibody fragment (16, 21, 22), which is linked to a composite CD28-CD3ζ signaling domain via a CD8α hinge region (17, 23, 24). NK-92 (kindly provided by Prof. Hans G. Klingemann) and NK-92/5.28.z cells were cultured in X-Vivo 10 medium with recombinant transferrin but without geneticin (Lonza, Basel, Switzerland), 5% heat-inactivated fresh frozen plasma (FFP) from blood group AB donors (DRK-Blutspendedienst Frankfurt am Main) and 100 IU/ml IL-2 (Proleukin S, Novartis) (15).

The aRMS cell line RH30 (*PAX3-FOXO1*) derived from a patient with alveolar RMS was purchased from DSMZ (Deutsche Sammlung von Mikroorganismen und Zellkulturen GmbH). Cells were cultured in Roswell Park Memorial Institute (RPMI) 1640 medium with GlutaMAX (Gibco) containing 10% heat-inactivated fetal bovine serum (FBS) (Sigma-Aldrich, St. Louis, MO, USA). For *in vivo* cell tracking, stable GFP/luciferase-expressing RH30 cells (RH30^GFP/luc+^) were generated by lentiviral transduction with the pSIEW-luc2 plasmid encoding enhanced green fluorescent protein (eGFP) and firefly luciferase linked by a T2A peptide (25). GFP-positive RH30^GFP/luc+^ cells were enriched by fluorescence-activated cell sorting using a FACSAria II instrument (BD Biosciences).

The RMS tumor organoids RMS102 (*PAX3-FOXO1*), RMS127 (*PAX3-FOXO1*), and RMS335 (*PAX7-FOXO1*) were generated from tumor biopsies of patients (**Table 1**) and laboriously cultured in complete culture medium BM1*, advanced DMEM/F12 (Gibco) with various growth factors, inhibitors and further additives, as published previously (27). For cell attachment, 0.1–0.5% basement membrane extract (R&D Systems) was added.

**Table 1:** Overview of RMS tumor cells used in this study.

Cell line/Tumor organoid	Origin	Disease instance of establishment	Histology	Karyotype/gene fusion state	Reference
RH30	Bone marrow metastasis 16-year-old male	Untreated primary disease	Alveolar	t(2,13) (*PAX3-FOXO1*), *TP53* mutation, amplification of 12q13–15 region including CDK4	(26)
RMS102	Lymph node metastasis (clavicle) of a 18-year-old male	Second relapse after vincristine, irinotecan, temozolomide treatment and radiotherapy (RT)	Alveolar	t(2,13) (*PAX3-FOXO1*)	(27)
RMS335	Lymph node metastasis (groin) of a 8-year-old female	Second relapse after vincristine, irinotecan, temozolomide treatment	Alveolar	t(1,13) (*PAX7-FOXO1*)	(27)
RMS127	Bone marrow metastasis of a 16-year-old male	Untreated primary disease	Alveolar	t(2,13) (*PAX3-FOXO1*)	(27)

### 2.2 Cell surface staining for ErbB2 in tumor organoid RMS cells

Tumor organoid RMS cells were stained with phycoerythrin (PE)-conjugated antibody (BioLegend, 324406) for ErbB2 and subsequently analyzed with a FACSCanto 10c flow cytometer (BD Biosciences). Cells were stained according to the manufacturer’s instructions. In brief, aliquots of 5x10^5^ cells were washed in phosphate-buffered saline (PBS) and subsequently stained with ErbB2 antibody for 20 minutes. After washing, 1x10^4^ events were recorded by flow cytometry using FACSDiva software (Version 6.1.3, BD Biosciences). Fluorescence minus one (FMO) controls were used as references. The actual ErbB2 receptor number was quantified using BD Quantibrite Beads (BD Biosciences) according to the manufacturer’s instructions. ErbB2 expression was determined using FlowJo Software (Version 10.8.1, Tree Star Inc.).

### 2.3 Cytotoxicity of NK-92/5.28.z cells against 2D tumor organoid RMS cells

The short-term toxicity of NK-92/5.28.z cells against tumor organoid RMS cells was determined using a europium release assay as reported elsewhere (19). In brief, the aRMS tumor cells were labeled with bis(acetoxymethyl) 2,2’:6’,2”-terpyridine-6,6”-dicarboxylate (BATDA) reagent (PerkinElmer) for 30 minutes. Thereafter, the cells were washed four times in probenecid (Sigma-Aldrich)-containing medium. Cell numbers were counted by Neubauer-improved hemocytometer. Cells were seeded into round-bottom 96-well plates at a seeding density of 5000 cells per well. Parental NK-92 or NK-92/5.28.z cells were added at effector to target (E:T) ratios ranging from 20:1 to 2.5:1. For maximum lysis, 20% Triton X-100 solution was added. After three hours of cocultivation, 20 µl of supernatant was transferred to another 96-well flat-bottom plate, and 200 µl of europium solution (PerkinElmer) was added. After an additional 15 minutes of incubation, the plates were measured with a Victor 3 1420 fluorometer (PerkinElmer). The specific lysis of the target cells was calculated by following formula:


$$\begin{array}{c} \%\;Specific\;lysis=\\ \left(\frac{experimental\;release\;\left(signal\right)-spontanous\;release\;\left(signal\right)}{maximum\;relase\;\left(signal\right)-spontanous\;release\;\left(signal\right)}\right)x100 \end{array}$$

### 2.4 Cytotoxic activity of NK-92/5.28.z cells against 3D aRMS tumor spheroids

The integrity of RH30 target and NK-92/5.28.z effector cells was verified. In brief, aliquots of 5x10^5^ cells were washed in phosphate-buffered saline (PBS) and subsequently stained with ErbB2 antibody or an IgG1κ isotype control (BioLegend, 400114) for 20 minutes according to the manufacturer’s instructions. After washing, 1x10^4^ events were recorded by flow cytometry using FACSDiva software (Version 6.1.3, BD Biosciences). In addition, ErbB2-CAR surface expression of NK-92/5.28.z cells was assessed. For this purpose 2x10^6^ either NK-92/5.28.z or NK-92 cells were washed once with DPBS and then incubated with Human TruStain FcX™ (Biolegend) for 20 minutes at 4°C. After another washing step in DPBS, the cells were incubated with 10 µg recombinant ErbB2 with human Fc tag for 20 minutes. After two additional washing steps, cells were stained with APC antibody directed against the Fc tag (Biolegend), 410712 cells, washed again, and analyzed using a BD FACS Canto 10c instrument (BD Biosciences).

RH30-derived aRMS spheroids were generated to assess the cytotoxic activity of NK-92/5.28.z cells to best mimic 3D tumor structures *in vitro*: tumor cells were resuspended and were counted by Neubauer-improved hemocytometer. 5000 RH30^GFP/luc+^ cells per well were seeded in 200 μl of RPMI +10% FBS into ultralow attachment 96-well round-bottom plates without prior coating (Corning) and spun down. On day four of culture, 100 µl of supernatant was carefully removed, and 1x10^5^ NK-92 or NK-92/5.28.z cells resuspended in 100 µl of NK-92 medium were added. Spheroids without effector cells were used as controls. Every three to four days, the medium was replaced by X-Vivo 10 medium with 5% FFP and 100 IU/ml IL-2. Spheroids were imaged on day 4, 5, 6, 8 and 10 after initial seeding using a Celigo Image Cytometer (Nexcelom Bioscience) with F-theta lens and AVT PIKE camera. The size of spheroids was quantified by the GFP signal using Fiji software (Version 2.3.0) (28).

### 2.5 Therapeutic activity of NK-92/5.28.z cells in a metastatic xenograft mouse model

*In vivo* experiments were approved by the appropriate government committee (Regierungspräsidium Darmstadt, Darmstadt, Germany; Gen.-Nr. TVA FK/1070) and were conducted according to the requirements of the German Animal Welfare Act.

Twenty-seven female 10- to 12-week-old nonobese diabetic (NOD)/severe combined immunodeficient (SCID)/Il2receptorgamma−/− (NSG) mice received sublethal irradiation with 2.5 Gy (Biobeam 2000) (d-1) according to the protocol for previously established metastatic RMS xenograft model closely resembling a clinical situation (29). One day later (d0), 1x10^5^ RH30^GFP/luc+^ cells resuspended in 100 µl of PBS were injected intravenously (iv) via the tail vein. Considering them as having an immanence risk for disease progression with then limited treatment options as previously shown (29), immunotherapies were applied iv one day after tumor cell injection (d+1) and were given six times in total over a period of four weeks. For analysis of the cytotoxic capacity of parental NK-92 and NK-92/5.28.z cells, mice were randomly divided into three different groups and injected with the following: control animals received medium (X-Vivo 10 medium with 5% FFP and 100 IU/ml IL-2, n=5), and treatment groups either received NK-92 cells (10x10^6^ cells each, n=12) or NK-92/5.28.z cells (10x10^6^ cells each, n=10). Both effector cell types were resuspended in X-Vivo 10 medium with 5% FFP and 100 IU/ml IL-2. Group size was set based on the experience from previous experiments with the same xenograft model.

Tumor growth was monitored weekly by bioluminescence imaging (BLI) using an IVIS Lumina II system (Perkin Elmer). Mice were anesthetized by isoflurane inhalation and received subcutaneous injections of 150 µg *in vivo* grade VivoGlo luciferin (Promega) in 100 µl of PBS per mouse. Fifteen minutes later, images were acquired in dorsal and ventral positions and subsequently analyzed by Living Image *In Vivo* Imaging Software (Perkin Elmer). Uniform regions of interest were used for all mice, and total flux (photon/s) was used for measurement. The signals of dorsal and ventral images were combined into a luminoscore, and statistical analysis of the tumor burden was performed by one-way ANOVA (30).

Altogether, tumor-bearing mice were randomly divided into the following three groups:

- Control: one course of six serial infusions with X-Vivo 10 media with 5% FFP and 100 IU/ml IL-2, n=5
- NK-92: 10x10^6^ cells, one course of six serial effector cell infusions, n=12
- NK-92/5.28.z: 10x10^6^ cells, one course of six serial effector cell infusions, n=10

During the experiment, animals were monitored daily for disease symptoms, xenogenic graft versus host disease (GVHD) and other adverse effects of the NK cell therapies for a maximum of 105 days. Mice with visible signs of disease progression, discomfort or physical abnormalities were painlessly sacrificed by isoflurane anesthesia followed by cervical dislocation.

#### 2.5.1 Preparation of single-cell suspensions from murine organs

Peripheral blood (PB), BM, lung, liver, gut and spleen samples were isolated and analyzed for persistence of human tumor or effector cells: BM was flushed out of the femur and tibia of mice. PB and BM were incubated with red blood cell lysis buffer (RBC Lysis Buffer (10x), BioLegend) according to the manufacturer´s instructions and washed once with PBS. The organs were cut in representative halves, and one half was preserved in formaldehyde for fluorescence microscopy. The other half was incubated with collagenase D solution (Roche, Basel, Switzerland), filtered through a 70 µm cell strainer, and washed with PBS. Aliquots of the cell suspensions were analyzed by flow cytometry and quantitative polymerase chain reaction (qPCR). Sample identity was blinded for the executors of the consecutively analyses.

#### 2.5.2 Flow cytometry

GFP expression of RH30^GFP/luc+^ was assessed shortly before use in experiments with a FACS Canto 10c device (BD Biosciences). CAR expression of NK-92/5.28.z cells was analyzed using a chimeric ErbB2-Fc protein (Sino Biological) after nonspecific Fc receptor blocking (Human TruStain FcX, BioLegend) and subsequent staining with an anti-IgG-Fc secondary antibody conjugated with allophycocyanin (APC) (BioLegend, 410712).

Cell samples from mouse organs were washed once in PBS and stained with phycoerythrin-cyanin 7 (PE/Cy7)-conjugated anti-human CD45 antibody (Biolegend, 304016).

Analyses were performed with FlowJo Software (Version 10.8.1, Tree Star).

#### 2.5.3 qPCR analysis of effector cells and tumor burden in murine organs

Genomic deoxyribonucleic acid (DNA) from murine organs was extracted using the Extractme Genomic DNA Kit (BLIRT S.A.). The percentage of human cells in the different murine tissues was determined by a quantitative real-time approach specifically amplifying the human albumin gene. In a second step, the proportions of NK-92 or NK-92/5.28.z cells and tumor cells within the human cell fraction were determined by a human-specific short tandem repeat (STR) genotyping approach. The tumor burden of each mouse was quantified in organs previously described as RMS metastatic sites (liver, lung, BM) (31, 32). For effector cell quantification, the spleen as a secondary lymphatic organ was also examined. Primers and probes were obtained from Eurofins (Eurofins Genomics), and the STR multiplex polymerase chain reaction (PCR) system Powerplex 16 (Promega) was used. Additionally, the presence of NK-92/5.28.z cells within the organs was verified by qPCR targeting the CD8α hinge region of the CAR construct.

#### 2.5.4 Histology and fluorescence microscopy

For histological analyses, organ sections were fixed in 4% buffered formalin, paraffin embedded, cut, and stained with hematoxylin-eosin (HE). Immunohistochemistry (IHC) antibodies targeting CD56, MYOD1, DESMIN and MYOGENIN were used to evaluate the RMS phenotype of the tumors. The stained tissue sections were rated by a pathologist for the expression of each protein (-, negative; +, low staining; ++, medium staining; +++, high staining).

Immunofluorescence staining was used to observe immune cell infiltration in organs and tumors: organ slices were deparaffinized and rehydrated. After antigen retrieval and a blocking step, primary mouse-anti targeting human CD45 antibody (Abcam, ab227741) and rabbit-against GFP antibody (Invitrogen, A11122) were added. For detection, secondary antibodies F(ab`)_2_ fragment goat-anti-mouse conjugated to Alexa 647 (Jackson ImmunoResearch, 115-606-003) and goat-anti-rabbit IgG conjugated to Alexa 488 (Invitrogen, A11008) were used, and nuclei were stained with 4´,6-eiamidino-2-phenylindole (DAPI) (Sigma-Aldrich). The localization of immune cells in the tumor tissue relative to blood vessels was determined by staining with rat-anti-mouse Meca32 antibody (BD Biosciences, 553849) and secondary goat-anti-rat Alexa546 antibody (Invitrogen, A-11081). The samples were examined with a BZ-X810 All-in-One Fluorescence microscope (Keyence) with basic lenses (CFI PlanApo λ 10x 0.45/4.00mm, CFI PlanApo λ 20x 0.75/1.00mm, CFI PlanApo λ 40x 0.95/0.25-0.16mm) and filter cubes Ex: 360/40 DM: 400 BA: 460/50 (DAPI), Ex: 470/40 DM: 495 BA: 525/50 (GFP), Ex: 620/60 DM: 660 BA: 700/75(Cy5).

In addition, the stained tissue sections with immune cell infiltration were analyzed by a pathologist for xenogenic GVHD.

### 2.6 Statistical analysis

For statistical analyses and graphical presentation, GraphPad Prism software (version 9, GraphPad Software, La Jolla, CA, USA) was used. The results are given as the mean ± standard deviation (SD). Differences between different groups were evaluated by two-tailed Student´s t test or one-way ANOVA using the Bonferroni-Dunn (nonparametric) method. Overall survivals are given as median with 95% confidence interval, Differences between the survival of different treatment groups were analyzed by the log-rank (Mantel-Cox) test. Differences with *p<* 0.05 (*), *p<* 0.01 (**), *p<* 0.001 (***) and *p<*0.0001 (****) were considered statistically significant.

## 3 Results

### 3.1 *In vitro* efficacy of NK-92/5.28.z cells against ErbB2-positive 2D tumor organoid RMS cells

**Figure 1:**
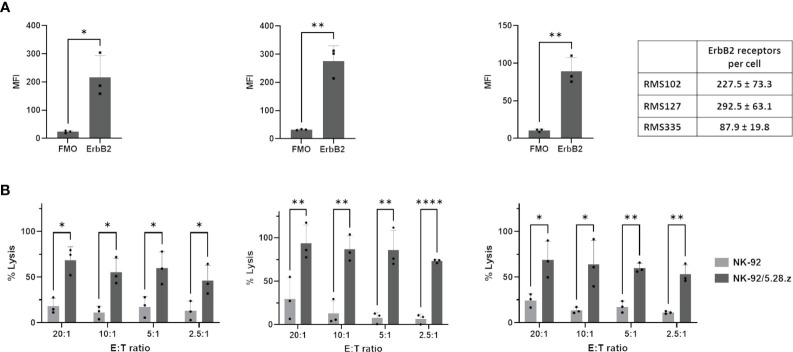
Lysis of ErbB2-positive, 2D patient-derived tumor organoid aRMS cells by parental NK-92 or NK-92/5.28.z cells. **(A)** ErbB2 surface expression on RMS102, RMS127 and RMS335 cells was confirmed by flow cytometry. Antigen density per cell quantified by flow cytometry is shown. **(B)** Cytotoxicity data of NK-92 or NK-92/5.28.z cells during short-term (3 hour) coculture with 2D patient-derived tumor organoid aRMS cells from europium release assays are given. Data of three independent experiments are shown as mean ± SD and differences were analyzed with a two-tailed Student´s t-test and were considered significant for p< 0.05 (*), p< 0.01 (**), p< 0.001 (****), p<0.0001 or ns.

NK-92/5.28.z cells represent a molecularly and functionally well-defined, ErbB2-specific single cell clone isolated upon transduction of NK-92 cells under GMP-compliant conditions with a lentiviral vector encoding a second-generation CAR directed against ErbB2 (15–17). In previous proof-of-concept analyses, we demonstrated the enhanced and CAR-specific cytotoxicity of NK-92/5.28.z cells compared to parental NK-92 cells against 2D ErbB2-positive RMS tumor cell lines (19). Here for *in vitro* assessment of efficacy, parental NK-92 or NK-92/5.28.z cells were tested against patient-derived tumor organoid aRMS cells in a 2D europium release assay. Maintaining actively growing rare patient-derived cells is challenging, but the tumor organoid RMS cells RMS102 (*PAX3-FOXO1*), RMS127 (*PAX3-FOXO1*) and RMS335 (*PAX7-FOXO1*) could be successfully passaged *in vitro* using previously published cultivation conditions (27). ErbB2 was expressed on all tumors and the actual receptor number was quantified (**Figure 1A**, 227.5 ± 73.3 receptors per cell (RMS102), 292.5 ± 63.1 (RMS127) and 87.9 ± 19.8 (RMS335)). *In vitro* cytotoxicity of NK-92/5.28.z cells was confirmed against tumor organoid aRMS cells. Of note, tumor cells were lysed to a high extent even at low E:T ratios of 5:1 and 2.5:1 and low ErbB2 surface expression of target cells by NK-92/5.28.z cells, but not by parental NK-92 cells (**Figure 1B**).

### 3.2 Cytotoxic activity of NK-92/5.28.z cells against 3D aRMS tumor spheroids

However, tumor cell suspensions or monolayers do not reflect the difficult-to-treat clinical situation of metastatic tumors. Tumor spheroids best represent the 3D structural, spatial heterogeneity and the hypoxia in the TME that suppresses antitumor immunity (33) and thus represent an intermediate between the preclinical *in vitro* and *in vivo* tumor metastasis models described below.

**Figure 2:**
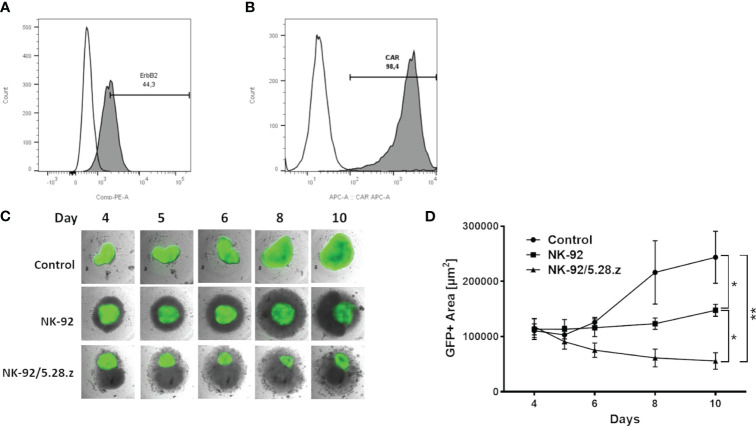
Cytotoxic capacity against ErbB2-positive 3D tumor spheroids. **(A)** ErbB2 surface expression on RH30 cells was confirmed by flow cytometry. **(B)** Expression of the anti-ErbB2-targeted CAR on NK-92/5.28.z cells was confirmed by flow cytometry. **(C)** Exemplary coculture images of NK-92 or NK-92/5.28.z cells added to established 3D RH30GFP/luc+ tumor spheroids on day 4 are shown. Imaging was performed on days 4, 5, 6, 8 and 10. **(D)** The areas with green fluorescent signals were quantified using Fiji. Data of three independent experiments are shown as mean ± SD. Differences were analyzed with a one-way ANOVA using the Bonferroni method and were considered significant for p< 0.05 (*), p< 0.01 (**) or ns.

For this ErbB2 expression of RH30 cells (**Figure 2A**) and CAR expression of NK-92/5.28.z cells (**Figure 2B**) was confirmed. The cytotoxic capacity of parental NK-92 and CAR-engineered NK-92.5.28.z cells was assessed against established, ErbB2-positive RH30^GFP/luc+^ tumor spheroids (**Figures 2C, D**). Coculture analyses were performed for an additional period of six days. NK-92/5.28.z cells inhibited tumor growth and effectively killed 3D cell structures. Furthermore, NK-92/5.28.z cells proliferated extensively, as shown by cell clustering, while in contact with tumor targets. In contrast, the proliferative and cytotoxic capacity of parental NK-92 cells was much less pronounced and the cells partially inhibited the growth of the 3D tumor spheroids. Spheroid size quantification showed significant reduced spheroid size after NK-92 cell immunotherapy compared to that of the untreated controls (*p =* 0.0351) on day 10 (**Figure 2D**). Of note, NK-92/5.28.z cell treatment led to significantly reduced spheroid size compared to untreated controls (*p* = 0.0013) and NK-92-treated spheroids (*p* = 0.0425) (**Figure 2D**). Comparable but less effective results were observed at lower E:T ratios (data not shown).

Altogether, NK-92/5.28.z cells, but not parental NK-92 cells effectively killed ErbB2-positive, 3D aRMS tumor spheroids *in vitro*.

NK-92/5.28.z cell line is a well-defined clinical grade NK-cell product, which may be provided as an third party, off-the-shelf product. However, because the cell line was derived from a 50-year-old patient with non-Hodgkin´s lymphoma, irradiation prior to infusion is mandatory for safety reasons. Here, irradiated NK-92/5.28.z cells showed inhibited proliferation (**Supplementary Figure 1A**), but retained their direct antitumor toxicity for at least 24 hours *in vitro* (**Supplementary Figure 1B**). We have previously shown that the therapeutic effects of non-irradiated and irradiated NK-92/5.28.z cells are comparable in mice (15, 16). Therefore, nonirradiated NK-92 and NK-92/5.28.z cells were used for *in vivo* assessments.

### 3.3 Therapeutic activity of NK-92/5.28.z cells in a metastatic RMS xenograft mouse model

**Figure 3:**
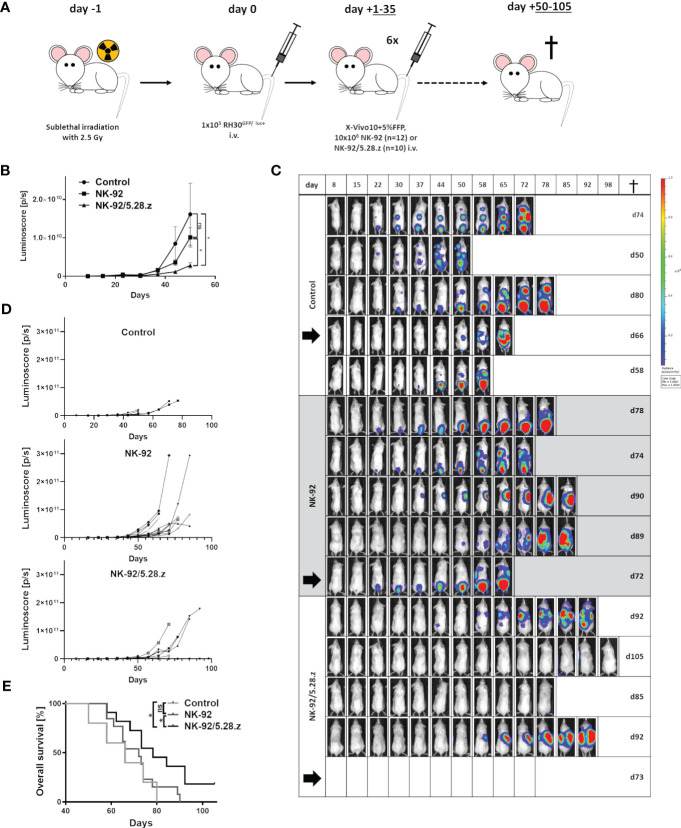
Therapeutic potential of NK-92 and NK-92/5.28.z cells in a metastatic xenograft NSG mouse model. **(A)** An experimental metastasis model in NSG mice carrying low tumor burden of a human aRMS (RH30GFP/luc+) xenograft was established to evaluate the therapeutic potential of NK-92 and NK-92/5.28.z cells *in vivo*. **(B–D)** Tumor development and growth was monitored and quantified by BLI combining both dorsal and ventral signals described by the luminoscore for each animal, shown by five representative examples. The mean ± SD is shown and differences were analyzed with a one-sided ANOVA using the Bonferroni-Dunn (nonparametric) method. Differences with p< 0.05 (*) were considered statistically significant. **(E)** The overall survival of untreated control (n=5), NK-92 cell-treated (n=12) and NK-92/5.28.z cell-treated (n=10) mice is shown. Differences between the survival of different treatment groups were analyzed by the log-rank (Mantel-Cox) test. Differences with p< 0.05 (*) were considered statistically significant. Black arrows indicate animals selected for exemplary images in **Figure 4A**.

Next, antitumor potential of serial injections of NK-92/5.28.z cells was assessed and the impact of the CAR was compared to parental NK-92 cells *in vivo*. Thereby, NSG mice carrying a metastatic aRMS (RH30^GFP/luc+^) xenograft were used to assess homing, tumor invasion, persistence, and antitumor effects as well as the xenogenic cytotoxicity (GVHD) of parental NK-92 and NK-92/5.28.z cells. Sequential infusions of effector cells were given preemptively during the low tumor burden period, and mice were followed for 105 days (end of experiment) (**Figure 3A**). In order to include all control mice in the BLI analyses, day 50 was chosen as the cutoff day for quantification of tumor engraftment assessed by BLI (**Figures 3B–D**). BLI confirmed reduced tumor size in the mice with NK-92/5.28.z cell treatment compared to the untreated (*p* = 0.0101) and NK-92-treated (*p* = 0.0159) animals at the time of first *in vivo* response assessment on day +50 (**Figures 3B–D**). In contrast, NK-92 cell immunotherapy showed no significant effect compared to that of the untreated controls (*p =* 0.2120) (**Figures 3C, D**).

Tumor engraftment in the controls without treatment was rapid. Life-limiting conditions occurred between 50 and 80 days after tumor cell inoculation (**Figure 3E**). NK-92 cell therapy (median survival, 72.5 ± 6.1 days) did not prolong survival to a statistically significant extent compared to that of the untreated controls (66.0 ± 13.4 days) (*p* = 0.501). In contrast, NK-92/5.28.z cell therapy (81.0 ± 8.8 days) significantly improved overall survival compared to that of the parental NK-92-treated (*p* = 0.0329) and untreated controls (*p* = 0.0366) (**Figure 3E**). Animals without symptoms were sacrificed 105 days after tumor cell injection. Of note, complete remission was observed in two out of ten NK-92/5.28.z-treated mice proving that this approach can be curative.

#### 3.3.1 Biodistribution, persistence, and *in vivo* cytotoxicity of NK-92/5.28.z cells

##### 3.3.1.1 Histology and immunofluorescence microscopy

Tumor engraftment and homing of effector cells at tumor sites was examined in livers, lungs, BM/bones, and spleens of mice. Organs of two animals per group were extracted and processed for histological analysis. An experienced pathologist rated the staining patterns of human aRMS (CD56, MYOD1, DESMIN and MYOGENIN). All detectable tumor lesions expressed CD56 (+++, membranous), MYOD1 (+++, nuclear), DESMIN (+, cytoplasmic) and MYOGENIN (++, nuclear).

**Figure 4:**
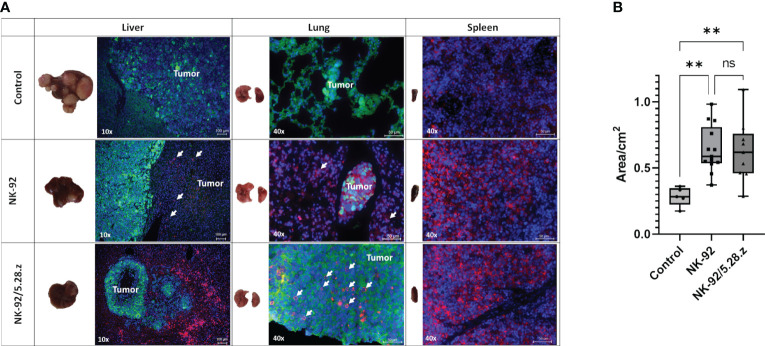
Macroscopic tumor lesions and immunofluorescence staining of metastatic tumors. **(A)** Tumor cells (Alexa 488, green) and immune cells (Alexa 647, red, in liver and lung additionally indicated by arrows) are shown; cell nuclei were stained with DAPI. Exemplary animals with tumor engraftment were selected from BLI analysis and were sacrificed on day 80 (control), day 72 (NK-92) and day 73 (NK-92/5.28.z), respectively (black arrows in **Figure 3D**). Immunofluorescence staining showed that NK-92 cells, and to a more pronounced extent NK-92/5.28.z cells, preferentially homed to spleens as well as to metastatic tumor sites in lung and liver, resulting in disseminated tumors (control), localized tumors (NK-92), and small tumor nodules (NK-92/5.28.z). Low level NK-92 and NK-92/5.28.z cell infiltration was observed within metastatic tumor sites. **(B)** The size of the spleen in each treatment group is shown. Differences were considered significant for p< 0.01 (**) or ns.

Tumor lesions were primarily observed in the livers and lungs of mice except in those with complete response to immunotherapy with NK-92/5.28.z cells. Multiple large (macroscopic) lesions were more likely to be present in the untreated and NK-92-treated mice vs. single small (microscopic) lesions in the NK-92/5.28.z-treated mice (**Figure 4A**). Immunofluorescence staining with an anti-hCD45 antibody revealed persistence of NK-92 and NK-92/5.28.z cells in the analyzed organs. Exemplary images of animals sacrificed on day 80 (control), day 72 (NK-92) and day 73 (NK-92/5.28.z) are shown (**Figure 4A**). Close proximity of immune and tumor cells illustrates the response to CAR-based therapy in our metastatic RMS model. Spleens of the mice with immune cell therapy were enlarged compared to the untreated controls (**Figure 4B**). Furthermore, all organs were scored regarding potential toxicity of the effector cells against normal tissues by an experienced pathologist. No tissue damage or tissue alterations through immune cell infiltration were detected.

**Figure 5:**
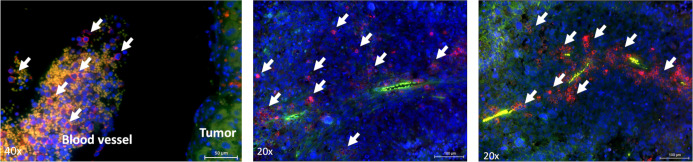
Localization of effector cells within the tumor tissue. Immunofluorescence staining of blood vessels (green) and NK-92/5.28.z cells or NK-92 cells (right image) (red, indicated by arrows) in liver tumors. Animals were sacrificed on day 72 or day 73.

Meca-32, a homodimeric glycoprotein, is primarily expressed on endothelial cells in mice. To better understand how immune cells reached and infiltrated tumors, we performed immunofluorescence staining of Meca-32, positive endothelial cells and hCD45 positive NK-92/5.28.z cells. We found colocalization of hCD45 positive effector cells with Meca-32 positive endothelial cells, suggesting the immune effector cells reach distant tumor sites via the circulatory system and extravasation, therewith augmenting their antimetastatic potential (**Figure 5**).

##### 3.3.1.2 Flow cytometry and qPCR

**Figure 6:**
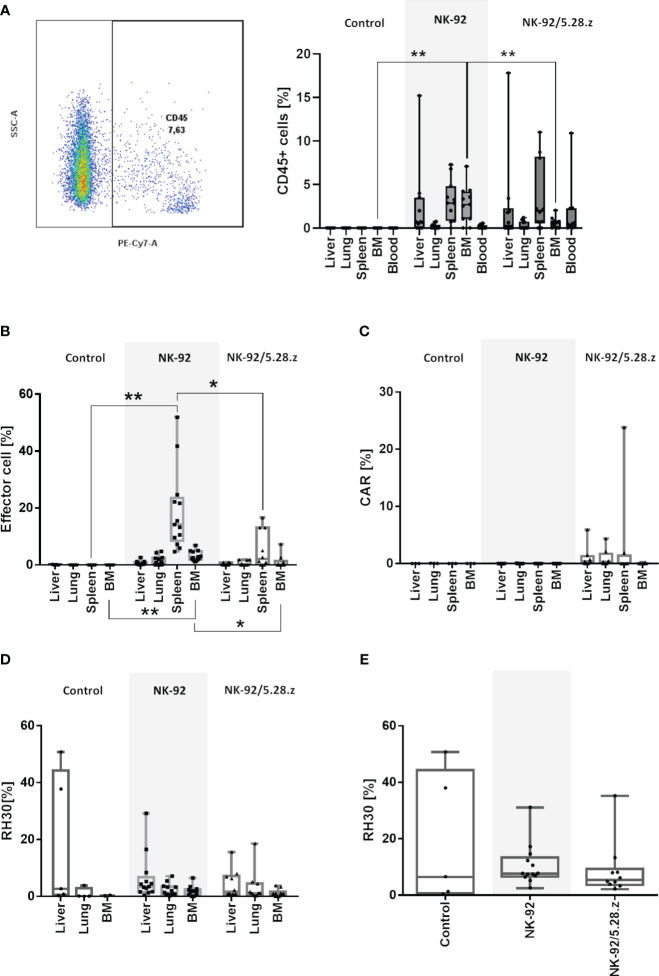
Homing and persistence of NK-92 and NK-92/5.28.z cells *in vivo*. Animals were sacrificed on day 50, 58, 66, 74 and 80 (control), day 58, 61, 65, 66, 72, 73, 74, 78, 89 and 90 (NK-92) and day 62, 68, 73, 78, 84, 85, 92 and 105 (NK-92/5.28.z). **(A)** Exemplary plot of human CD45 positive NK-92 and NK-92/5.28.z cells, detectable in all analyzed treatment tissue samples by flow cytometry. **(B–D)** Human immune cells **(B)**, NK-92/5.28.z cells **(C)** and aRMS cells **(D)** were detected by qPCR and are shown as boxplots for different organs of the control (n=5), NK-92 cells (n=12), and NK-92/5.28.5 cells (n=10). **(E)** The overall tumor burden was calculated for each animal. Differences were analyzed with a one-way ANOVA using the Bonferroni method and were considered significant for p< 0.05 (*), p< 0.01 (**) or ns (not labled).

The persistence of NK-92 and NK-92/5.28.z cells at tumor sites was further confirmed by flow cytometry using human anti-CD45 antibody staining (**Figure 6A**). In the treatment groups, immune effector cells were detected in all analyzed organs, including the blood. However, cells were too low in numbers to perform flow cytometry analyses for surface CAR expression. These results were further confirmed by qPCR. High immune effector cell infiltration was shown in secondary lymphoid organs such as the spleen (**Figure 6B**). Of note, the persistence of immune effector cells in spleens was more pronounced in the NK-92-treated mice than in the NK-92/5.28.z-treated mice. In addition, qPCR of the tissues detecting the presence of the CD8α linker of the CAR showed that NK-92/5.28.z cells were still detectable, but again to low in numbers to perform flow cytometry analyses for surface CAR expression (**Figure 6C**).

Quantifiable tumor levels were detected by qPCR (**Figures 6D, E**). The number of tumor cells as well as overall tumor burden was highest in liver tissues followed by lung tissues and was lowest in the BM. High tumor levels were more likely to be present in the untreated and NK-92-treated mice than in mice treated with NK-92/5.28.z cell therapy. Due to the strong autofluorescence properties of analyzed organs, which interfered with flow cytometry emission signals, number of remaining RMS cells and ErbB2 surface expression was not assessable by flow cytometry.

## 4 Discussion

Severe CRS and/or ICANS, modest antitumor activity, antigen escape, restricted trafficking, and limited tumor infiltration due to the host and/or the TME and bulky diseases are limitations of CAR-T therapy in solid tumors (9, 34). A group at Baylor College of Medicine, Houston, Texas, USA, reported safety and a durable remission over more than four years after initiating ErbB2-CAR T-cell infusions in combination with checkpoint inhibition to overcome the immunosuppressive TME in a child with metastatic aRMS (14).

Autologous CAR-T cells are made from the cancer patients’ own peripheral blood lymphocytes, therefore transduction efficiency, T-cell subtype distribution, and activation state can vary, affecting overall product composition, quality, and toxicity. We suggest that serial administrations of CAR-engineered innate immune cells such as the NK-92 cell line may translate into a feasible, safe, effective and affordable treatment strategy for r/r metastatic aRMS. In a proof-of-concept analysis, we previously showed that the CAR-engineered cell line NK-92/5.28.z has strong *in vitro* cytotoxicity against ErbB2-positive tumor cell lines, including aRMS (19). Thereby, the intrinsic natural and immunomodulatory cytotoxicity of NK-92 cells was further enhanced through the expression of the appropriate CAR. Intrinsic cytotoxicity is mainly based on signaling through activating NK cell receptors and is less likely to allow tumor escape by antigen loss as may occur in the case of major histocompatibility complex (MHC)-mediated killing by T cells (35). Therefore, NK-92/5.28.z cells harbor additional antitumor capacity against r/r malignancies. Cytokines and chemokines released by activated NK-92/5.28.z cells interferon (INF)-γ, granulocyte macrophage-colony stimulating factor (GM-GSF) and tumor necrosis factor (TNF)-α), which are essential signals of both innate and adoptive immunity, are likely to support the antitumor toxicity of bystander cells *in vivo* but are less likely to induce severe CRS or ICANS upon target engagement than CAR-T cells (17, 19, 36, 37). Unlike CAR-T cells, NK-92/5.28.z cells may be used as a third-party, ready-to-use, off-the-shelf product. Indeed, the alloreactivity of NK-92 cell-centered cell therapy is known to be low even across human leucocyte antigen barriers (38–41). Therefore we propose NK-92/5.28.z cells as a consolidation treatment for children and young adults with r/r, metastatic ErbB2-positive RMS tumors after re-induction radiotherapy/chemotherapy.

Previous studies showed selective recognition and elimination of ErbB2-positive breast carcinoma (15) and glioblastoma (16) cells with high target antigen expression levels by NK-92/5.28.z cells. Despite low and heterogeneous ErbB2 expression levels, we confirmed sufficient and ErbB2-specific antitumor capacity of NK-92/5.28.z cells against both, r/r 2D patient-derived tumor organoid RMS cells and 3D RMS tumor spheroid models. Here, susceptibility to lysis of NK-92/5.28.z cells was not exclusively related to surface ErbB2 expression of target cells. However, good accessibility of the target antigen on tumor organoid RMS cells and tumor spheroids for NK-92/5.28.z cells led to efficient *in vitro* stimulation, activation and expansion of the cells. Sufficient infiltration into the TME of 3D tumor spheroids further rendered NK-92/5.28.z cells fit and responsive without the tumor cells evading NK-92/5.28.z cell cytotoxicity redirected toward a single tumor antigen.

The *in vivo* antitumor activity of sequential infusions of parental NK-92 and CAR-engineered NK-92/5.28.z cells was evaluated in an experimental metastasis model in NSG mice carrying an aRMS xenograft. We confirmed that both the metastatic behavior and the histopathological characteristics of the *PAX3-FOXO1* positive human aRMS cell line RH30^GFP/luc+^ were retained *in vivo*. Compared to the control and treatment with parental NK-92 cells, sequential infusions of NK-92/5.28.z cells significantly prolonged survival, suppressed tumor development and tumor growth and inhibited tumor engraftment in two out of ten mice. Analyses of tumor-targeted organs by PCR revealed the lowest tumor burden in the NK-92/5.28.z cell-treated animals underscoring the response without immune evasion to CAR-based therapy in metastatic RMS. The *in vivo* trafficking and biodistribution of both NK-92 and NK-92/5.28.z cells were confirmed by flow cytometry, IHC and PCR in various tissue samples, including secondary lymphoid organs such as spleens. Close proximity of NK-92 and NK-92/5.28.z cells to tumor sites in livers and lungs as well as infiltration via the circulatory system was determined by fluorescence microscopy analysis. Detection of NK-92 and NK-92/5.28.z cells suggests intrinsic and CAR-specific immune responses that enable activation, expansion, and persistence of the effector cells *in vivo*.

The immune infiltration into the TME of aRMS tumors is controversial. The hypoxic conditions of growing tumors are associated with the emergence of resistant tumor cell clones and immune evasion (42). Accordingly, the absence of T and B cells and the presence of an extracellular matrix (ECM) and cancer-associated fibroblasts was reported by different groups (43, 44). In contrast to embryonal RMS, CD54-positive microvessels are almost absent in aRMS, resulting in low numbers of tumor-infiltrating T cells and M1 macrophages. This phenomenon is considered one potential reason for the poor prognosis of immunologically cold alveolar subtype RMS (44). Recently published single-cell transcriptomic data indicate higher numbers of T and NK cells, but also of M2 macrophages within the TME, that may support immune escape (45). The lack or suppression of proinflammatory immune cell infiltration suggests that the ECM and hypoxic and acidic conditions of the TME may also hamper adoptive cellular immunotherapy of metastatic aRMS (46, 47). Our data indicate that NK-92/5.28.z cells are able to overcome the TME barrier in both preclinical *in vitro* and *in vivo* models. As immunodeficient mice were used for *in vivo* experiments, only the intrinsic, i.e., the CAR-mediated and natural cytotoxic but not the immunomodulatory capacities of NK-92/5.28.z cells were assessed in this study. However, neither immunocompetent humanized NSG mouse models nor PDX models can fully mimic the *in vivo* human immune system as well as the immunosuppressive TME and even more will hamper disseminated tumor engraftment in mice. Previous reports indicated crosstalk between CAR-NK and host dendritic cells, resulting in the induction of endogenous adaptive antitumor immune responses and immunological memory upon NK-92/5.28.z cell treatment of tumor-bearing immunocompetent animals (16, 21). This immunomodulatory capacity that may lead to activation of the adaptive immune system in addition to the intrinsic and CAR-mediated cytotoxicity of NK-92/5.28.z cells is an important aspect when considering systemic and repetitive application of irradiated NK-92/5.28.z cells for the treatment of patients. Neither NK-92 cells nor NK-92/5.28.z cells displayed significant toxicity, indicated by the absence of symptoms of CRS, xenogenic GVHD, or histological damage in GVHD- and tumor-targeted organs, sometimes seen against tumor cells with low-level antigen expression. Nevertheless, for safety reasons irradiation of human NK-92/5.28.z cell line derived from a 50-year-old patient with non-Hodgkin´s lymphoma is mandatory. Irradiation with 10 Gy resulted in a limited life span and expansion potential thereby providing safety but not reduced anti-RMS cytotoxic capacity of NK-92/5.28.z cells within 24 hours thereafter (17, 48).

In summary, our data show that NK-92/5.28.z cells exhibit high cytotoxicity in r/r ErbB2-positive 2D patient-derived tumor organoid RMS cells and 3D aRMS tumor spheroid models *in vitro*, which was reproduced and confirmed in an experimental metastasis model in NSG mice *in vivo* not representing the adoptive immunity of bystander cells. NK-92/5.28.z cells were capable of migrating toward and persisting at tumor sites. As long as serial infusions of NK-92/5.28.z cells were applied, tumor growth and development were inhibited, which resulted in increased survival of the mice. Furthermore, tumors again responded to a second course of NK-92/5.28.z cell therapy, suggesting that no treatment-induced antigen loss occurred (data not shown). *In vivo* treatment of mice with parental NK-92 cells had no significant effect, indicating that the observed therapeutic efficacy of NK-92/5.28.z cells was mainly mediated by the second-generation ErbB2-CAR construct.

NK-92/5.28.z cell therapy is already under safety evaluation in patients with recurrent glioblastoma, which is an advantage when considering to extend clinical application of this ready-to-use CAR-engineered product to other cancer indications such as metastatic aRMS. We therefore propose to use NK-92/5.28.z cells for consolidation therapy of r/r, metastatic ErbB2-positive RMS following re-induction radiotherapy/chemotherapy to benefit from their multiple endogenous cytolytic pathways in addition to direct CAR-mediated killing. Thereby, the limited life-span and lack of *in vivo* expansion of NK-92/5.28.z cells upon irradiation may be addressed by repeated dosing and combination with checkpoint inhibition or other anticancer therapies that could further enhance or benefit from the immunomodulatory activity of the cells.

## Data availability statement

The raw data supporting the conclusions of this article will be made available by the authors, without undue reservation.

## Ethics statement

The animal study was approved by Regierungspräsidium Darmstadt, Darmstadt, Germany; Gen.-Nr. TVA FK/1070. The study was conducted in accordance with the local legislation and institutional requirements.

## Author contributions

CH, LM, WW, EU, HB, MM and ER conceived and designed the experiments. CH, LM, HK, HB, EG, EU, MM and ER performed the experiments. CH, LM, HK, HB, EU, MM and ER analyzed the data. HK, HB, TT, WW, EG, EU, MM, MK, FH, JD, J-HK, PB contributed to reagents, materials and analysis tools. CH, LMM and ER wrote the manuscript. HK, HB, TT, WW, EG, EU, MM, M, FH, JD, J-HK, PB and MM revised the manuscript. J-HK and PB supervised the research. All authors contributed to the article and approved the submitted version.
