# Supplementary material for: ErbB2 (HER2)-CAR-NK-92 cells for enhanced immunotherapy of metastatic fusion-driven alveolar rhabdomyosarcoma
Source: Front Immunol. 2023 Aug 18;14:1228894. doi: 10.3389/fimmu.2023.1228894 (PMC10471977; doi:10.3389/fimmu.2023.1228894)
Supplement: Supplementary file 1 [file DataSheet_1.pdf]

Supplementary Material

ErbB2 (HER2)-CAR-NK-92 cells for enhanced immunotherapy of metastatic fusion-driven alveolar rhabdomyosarcoma

Catrin Heim[1], Laura M Moser[1,2,3,4], Herman Kreyenberg[1], Halvard B. Bonig[5,6], Torsten Tonn[7,8], Winfried S Wels[9,2,3], Elise Gradhand[10,4], Evelyn Ullrich[11,2,3,4], Michael T Meister[12,13], Marian Groot Koerkamp[12,13], Frank C.P. Holstege[12,14], Jarno Drost[12,13], Jan-Henning Klusmann[15,2,3,4], Peter Bader[1,4], Michael Merker[1,4†], Eva Rettinger*[1,2,3,4†]

*** Correspondence:** Eva Rettinger, eva.rettinger@kgu.de

†These authors contributed equally to this work and share last authorship

Supplementary Figures

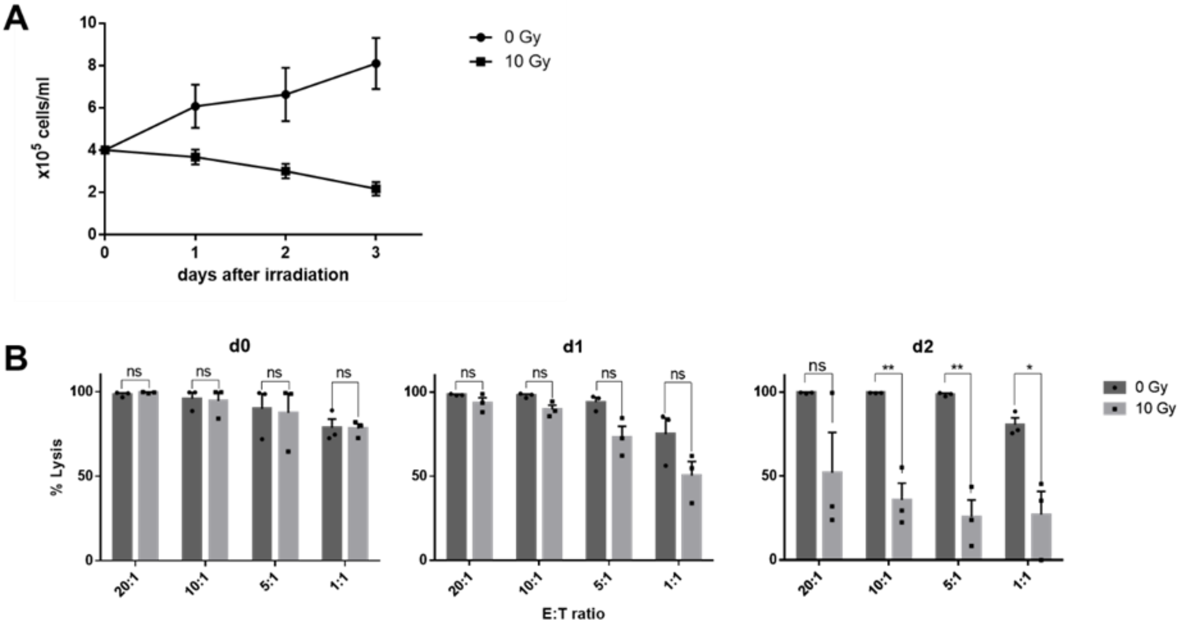

Supplementary Figure 1. Sensitivity of NK-92/5.28.z cells to γ-irradiation. (A) Proliferative capacity of NK-92/5.28.z cells after γ-irradiation at 10 Gy is shown. (B) Antitumor activity was assessed by luciferase-based cytotoxicity assays during 18 h cocultures with RH30 cells on day 0, 1 and 2 after γ-irradiation. Data of three independent experiments are shown as mean ± SD. Differences were analyzed with a two-tailed Student´s t-test and were considered significant for $p < 0.05$ (*), $p < 0.01$ (**), $p < 0,005$ (***) or ns.
